# Adjunctive Magnetic Seizure Therapy for Schizophrenia: A Systematic Review

**DOI:** 10.3389/fpsyt.2021.813590

**Published:** 2022-01-10

**Authors:** Xin-Yang Zhang, Huo-Di Chen, Wan-Nian Liang, Xin-Hu Yang, Dong-Bin Cai, Xiong Huang, Xing-Bing Huang, Cheng-Yi Liu, Wei Zheng

**Affiliations:** ^1^The Affiliated Brain Hospital of Guangzhou Medical University (Guangzhou Huiai Hospital), Guangzhou, China; ^2^Laboratory of Laser Sports Medicine, School of Sports Science, South China Normal University, Guangzhou, China; ^3^Guangdong Teachers College of Foreign Language and Arts, Guangzhou, China; ^4^Wanke School of Public Health, Tsinghua University, Beijing, China; ^5^Shenzhen Traditional Chinese Medicine Hospital, Shenzhen, China

**Keywords:** magnetic seizure therapy, schizophrenia, systematic review, neurocognitive function, response

## Abstract

**Objective:** The efficacy and safety of adjunctive magnetic seizure therapy (MST) for patients with schizophrenia are unclear. This systematic review was conducted to examine the efficacy and safety of adjunctive MST for schizophrenia.

**Methods:** Chinese (WanFang and Chinese Journal Net) and English (PubMed, EMBASE, PsycINFO, and the Cochrane Library) databases were systematically searched.

**Results:** Two open-label self-controlled studies (*n* = 16) were included and analyzed in this review. In these studies, the Positive and Negative Syndrome Scale (PANSS) total scores and Brief Psychiatric Rating Scale (BPRS) total scores significantly decreased from baseline to post-MST (all *Ps* < 0.05), without serious adverse neurocognitive effects. Mixed findings on the neurocognitive effects of adjunctive MST for schizophrenia were reported in the two studies. A discontinuation rate of treatment of up to 50% (4/8) was reported in both studies. The rate of adverse drug reactions (ADRs) was evaluated in only one study, where the most common ADRs were found to be dizziness (25%, 2/8) and subjective memory loss (12.5%, 1/8).

**Conclusion:** There is inconsistent evidence for MST-related adverse neurocognitive effects and preliminary evidence for the alleviation of psychotic symptoms in schizophrenia.

## Introduction

Schizophrenia is a severely disabling psychiatric disorder affecting ~1% of the population worldwide (1–3). The economic burden of schizophrenia amounted to $155.7 billion in the United States in 2013 (4). Despite advances in psychopharmacologic therapy, nearly 50% of schizophrenia patients do not respond to therapy with antipsychotics (5–7). Consequently, non-pharmacological therapies, such as augmentation strategies, have been widely used for schizophrenia in clinical practice, with neuromodulation techniques being particularly common (8), including electroconvulsive therapy (ECT) (9–11), repetitive transcranial magnetic stimulation (rTMS) (12), deep brain stimulation (DBS) (13, 14), non-convulsive electrotherapy (15, 16), transcranial direct current stimulation (tDCS) (17–19), and magnetic seizure therapy (MST) (20, 21).

ECT is the most effective treatment for individuals suffering from schizophrenia (22, 23) and mood disorders (24). For example, a recent randomized controlled trial (RCT) (22) and meta-analysis (23) found that the augmentation of clozapine with ECT is a highly effective therapy for clozapine-resistant schizophrenia (CRS). ECT is also an effective and safe method in treating elderly patients with treatment-resistant depression (TRD) (25). Interestingly, as reported by Osler et al.'s study (26), ECT was related to a decreased rate of dementia in patients aged 70 years and older. However, ECT-related adverse neurocognitive effects, including disorientation, amnesia, and executive dysfunction, prevent the use of ECT as a first-choice therapy for schizophrenia and mood disorders (27–29). Importantly, the damaging stigma surrounding ECT also potentially impedes widespread acceptance of this therapy among individuals suffering from schizophrenia (30).

MST is a novel neurotherapeutic intervention that induces therapeutic seizures based on high-frequency rTMS (31–34). MST appears to have a favorable clinical benefit on neurocognitive adverse effects and thus has been proposed as an alternative to ECT (21, 35). Accumulating evidence shows that MST is associated with relatively fewer neurocognitive adverse effects than ECT for major depressive disorder (MDD) (36, 37). In a recent meta-analysis, MST was associated with shorter recovery and reorientation times and lower cognitive impairment for MDD than ECT (38). However, inconsistent findings have been reported in two studies on patients with schizophrenia receiving MST treatments (20, 21).

To date, no systematic review on the efficacy and safety of adjunctive MST for schizophrenia has been published. Therefore, the target of the current study was to investigate the efficacy and safety of MST as an adjunctive therapy in schizophrenia.

## Methods

### Eligibility Criteria

This systematic review was conducted according to PRISMA guidelines (39). Studies were selected and screened for inclusion in line with the following ***PICOS*** criteria. ***P***articipants: adult subjects with a diagnosis of schizophrenia based on any standardized diagnostic instruments. ***I***ntervention vs. ***C***omparison: treatment as usual (TAU) plus MST vs. TAU plus ECT (RCTs); MST added to TAU (open-label prospective trials). ***O***utcomes: in this systematic review, the primary outcome was the improvement of psychotic symptoms, as measured by the Positive and Negative Syndrome Scale (PANSS) (40) or Brief Psychiatric Rating Scale (BPRS) (41). Key secondary outcomes were adverse neurocognitive effects, study defined response and remission, the rate of adverse drug reactions (ADRs), and discontinuation of treatment for any reason. ***S***tudy: only published case series, open-label prospective trials or RCTs examining the efficacy and safety of adjunctive MST for individuals experiencing schizophrenia were eligible for inclusion. Meta-analyses and systematic reviews were excluded.

### Study Selection

Two investigators (XYZ and XHY) independently searched English (PubMed, EMBASE, PsycINFO, and Cochrane Library) and Chinese (WanFang and Chinese Journal Net) databases from the date of inception until October 6, 2021 for studies on adjunctive MST for schizophrenia using the following search terms: (“magnetic seizure therapy”[Mesh] OR magnetic seizure therapy OR MST) AND (“schizophrenia”[Mesh] OR schizophrenic disorder OR disorder, schizophrenic OR schizophrenic disorders OR schizophrenia OR dementia praecox). Similarly, two independent investigators (XYZ and XHY) evaluated whether the potentially relevant studies fulfilled the inclusion criteria of this systematic review, and the senior author (WZ) was consulted in case of any differences of opinion.

### Data Extraction and Assessment of Study Quality

Two investigators (XYZ and XHY) independently extracted data from each included study. Any discrepancies in data entry between the two investigators (XYZ and XHY) were discussed, and the senior author (WZ) was consulted as needed. We contacted the first and/or corresponding authors to acquire any missing information as necessary. The quality of each included RCT and open-label prospective trial was evaluated by two independent investigators (XYZ and XHY) using the Cochrane risk of bias (42) and the Newcastle-Ottawa Scale (NOS), respectively (43). A NOS score of 7 or above was considered high quality. The quality of evidence and strength of recommendations of this systematic review was evaluated using the grading of recommendations assessment, development, and evaluation (GRADE) system (44), ranging from “very low quality,” “low quality,” “moderate quality” to “high quality.

## Results

### Literature Search

**Figure 1:**
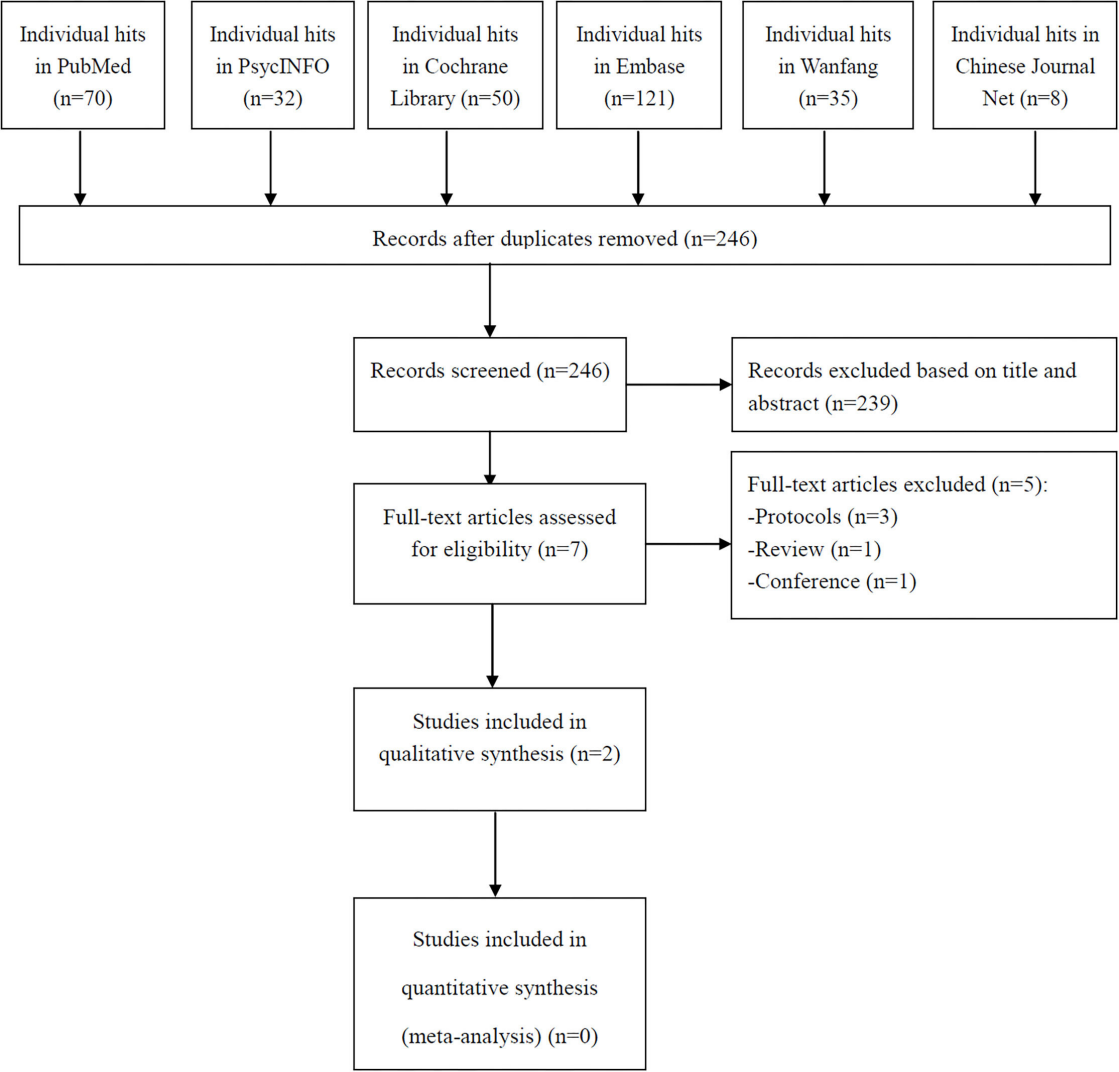
PRISMA flow diagram.

As shown in Figure 1, a total of 316 hits were identified from the aforementioned databases. Finally, two open-label self-controlled studies met the inclusion criteria of this systematic review (20, 21). It was not possible to conduct a meta-analysis because of the inconsistencies in study methodologies, parameters of MST, and antipsychotic dosages.

### Characteristics of Included Studies

The characteristics of the two open-label self-controlled studies (*n* = 16) (20, 21) are summarized in Table 1. The included studies were published within the last 3 years, showing that adjunctive MST for schizophrenia is a new clinically important topic. One study was conducted in China (20), and the other was conducted in Canada (21). The studies differed in that the MST was administered using a stimulator machine at a fixed frequency of 25 Hz (100% output) in Jiang et al.'s study (20) and a flexible frequency of 25–100 Hz (100% output) in Tang et al.'s study (21).

**Table 1:** Summary of characteristics of included studies.

**Study (country)**	***N* (♂/♀)**	**Diagnosis (%)**	**Diagnostic criteria**	**Age: yrs (range)**	**Duration of illness (yrs)**	**- Design** **- MST device**	**- Output** **- Frequency**	**Anesthesia (mg/kg)**	**Treatment duration (sessions/** **wks)**	**Number of treatment (sessions)**	**NOS scores**
Tang et al. (21) (Canada)	8 (7/1)	SCZ (75%) and SCZ-A (25%)	DSM-IV	45.9 (18–65)	24.9	-Open-label -MagPro MST, MagVenture	-100%−25 to 100 Hz	Methohexital sodium^a^ (0.375–0.75 mg/kg)	2–3	15.6 (range: 6–24)	7
Jiang et al. (20) (China)	8 (3/5)	SCZ (100%)	DSM-5	25.3 (18–55)	5.6	-Open-label -MagVenture A/S, Denmark	-100%−25 Hz	Etomidate (0.21–0.3 mg/kg) and propofol (1.82–2.44 mg/kg).	2–3	7.4 (range: 1–10)	7

*^a^If a trained psychiatrist diagnosed the patient as having inadequate control of seizures, the dose of methohexital was decreased and remifentanil (1.0–1.5 μg/kg) was used as a second anesthetic agent for convulsive therapy.*

*DSM-5, Diagnostic and Statistical Manual of Mental Disorders, 5th edition; DSM-IV, Diagnostic and Statistical Manual of Mental Disorders, 4th version; MST, magnetic seizure therapy; NOS, Newcastle-Ottawa Scale; N, number of patients; SCZ, schizophrenia; SCZ-A, schizoaffective disorder; wks, weeks; yrs, years*.

### Quality Assessment

The Cochrane risk of bias was not used because no RCTs were included in this systematic review. The NOS scores of the two self-controlled studies (20, 21) were 7 points (high quality) (Table 1). Following the GRADE system, the quality of evidence for each outcome was considered as “low” (Supplementary Table 1).

### Psychotic Symptoms

As shown in Table 2, patients with schizophrenia experienced a significant improvement in psychotic symptoms post-MST, as measured by the PANSS scale (total scores and positive subscale scores) (20) and the BPRS scale (total scores) (21) (all *Ps*<*0.05*). In Jiang et al.'s study (20), 3 out of 8 patients (37.5%) responded to MST. In Tang et al.'s study (21), 37.5% (3/8) of the patients met the remission criteria, and 50% (4/8) of the patients met the response criteria.

**Table 2:** The improvement of psychotic symptoms after MST.

**Study**	**PANSS/BPRS**	**Pre-MST (mean ± SD, *n*)**	**Post-MST (mean ± SD, *n*)**	***P-value***
Tang et al. (21)	Completers: BPRS total scores	40.5 ± 1.0 (4)	25.5 ± 4.4 (4)	**0.008**
	All subjects: BPRS total scores	42.6 ± 4.4 (8)	32.4 ± 8.9 (8)	**0.018**
Jiang et al. (20)	PANSS total scores	97.3 ± 10.0 (8)	71.5 ± 22.4 (6)	**<0.05**
	PANSS positive scores	66.4 ± 20.6 (8)	63.7 ± 22.0 (6)	**<0.05**

*Bolded values are P < 0.05.*

*BPRS, Brief Psychiatric Rating Scale; MST, Magnetic Seizure Therapy; n, number of patients; PANSS, Positive and Negative Syndrome Scale*.

### Neurocognitive Functions

Table 3 summarizes the neurocognitive effects of adjunctive MST for schizophrenia. Jiang et al. found using the Repeatable Battery for the Assessment of Neuropsychological Status (RBANS) that MST was associated with an improvement in immediate memory (66.7%, 2/3) but not in delayed memory (20). In the other study, MST was found to produce an significant decrease in neurocognitive performance, as measured by the Autobiographical Memory Inventory Short Form (AMI-SF) (*P* < 0.05), but no such decrease was found using the MATRICS Consensus Cognitive Battery (MCCB), Trail Making Test (TMT), Stroop Test or Verbal Fluency using the Controlled Oral Word Association Test (COWAT), and Montreal Cognitive Assessment (MoCA) (all *Ps* > 0.05) (21).

**Table 3:** Neurocognitive adverse events after MST.

**Study**	**Neurocognitive domains**	**Measure**	***N***	**Mean change^**a**^**	**SD**	***P-value***
Tang et al. (21)	Autobiographical memory speed of processing	AMI-SF	5	9.8	4.0	**0.005**
		BACS SC	5	1.2	8.0	0.755
		Fluency	5	3.8	10.7	0.471
		TMT-A	5	6.4	10.5	0.243
	Working memory non-verbal	Spatial span^b^	5	5.8	7.9	0.177
	Working memory verbal	LNS	4	0.8	9.3	0.882
	Verbal learning	HVLT-R	5	2.6	8.3	0.521
	Visual learning	BVMT-R	5	2.8	11.2	0.607
	Reasoning and problem solving	Mazes^c^	5	4.8	7.4	0.220
	Cognitive set-shifting	TMT-B	3	15.0	15.5	0.236
	Processing speed and inhibition	Stroop	5	12.4	21.3	0.263
	Verbal fluency	COWAT	5	8.4	9.0	0.105
	Mild cognitive impairment	MoCA	5	1.8	2.1	0.090
	**Neurocognitive domains**	**Measure**	***N***	**Pre-MST (mean)**	**Post**-**MST (mean)**	***P-value***
Jiang et al. (20)	Immediate memory	RBANS	3	58.0	68.0	NR
	Delayed memory	RBANS	3	54.0	66.0	NR

*^a^Mean change: post-MST scores minus pre-MST scores.*

*^b^Spatial Span from the Weschler Memory Scale-third edition.*

*^c^Mazes from Neuropsychological Assessment Battery.*

*Bolded values are P < 0.05. AMI-SF, Autobiographical Memory Inventory Short Form; BVMT-R, Brief Visuospatial Memory Test-Revised; BACS SC, Brief Assessment of Cognition in Schizophrenia Symbol Coding; COWAT, Controlled Oral Word Association Test; HVLT-R, Hopkins Verbal Learning Test Revised; LNS, letter-number span; MoCA, Montreal Cognitive Assessment; NR, not reported; N, number of patients; RBANS, Repeatable Battery for the Assessment of Neuropsychological Status; TMT-A, Trail Making Test Part A; TMT-B, Trail Making Test Part B*.

### Discontinuation and ADRs

In both studies, discontinuation of MST for any reason was reported for 50% (4/8) of the participants (20, 21). The patients' subjective experience of MST was only evaluated in one study, and the most common ADRs were found to be dizziness (25%, 2/8) and subjective memory loss (12.5%, 1/8) (20).

## Discussion

This article is the first systematic review on the efficacy and safety of MST as an adjunctive therapy for schizophrenia. Only two open-label self-controlled studies (20, 21) were included in this systematic review, corresponding to a total of 16 patients. The main findings were that adjunctive MST was efficacious for total psychopathology in schizophrenia, as measured by the PANSS and the BPRS, and did not have serious adverse neurocognitive effects. Both studies examined the neurocognitive effects of adjunctive MST for schizophrenia, but mixed findings were reported. A relatively high rate of discontinuation of MST for any reason was reported in both studies. The most common ADRs were evaluated in only one study and found to be dizziness and subjective memory loss (20). Although MST appears to be an interesting and potentially important adjunctive therapy for patients suffering from schizophrenia, these findings should be clearly verified in future studies with a randomized double-blind ECT-controlled design.

This systematic review shows there is preliminary evidence for the antipsychotic effects of MST in schizophrenia and negligible neurocognitive adverse effects. As reported in the two included studies (20, 21), the response rate of adjunctive MST for individuals experiencing schizophrenia ranged from 37.5 to 50%, which was far lower than the reported response rate to ECT of up to 74% (45). However, Kayser et al. reported that up to 69% of patients with TRD responded to MST (46). The latest meta-analysis (10 studies, 285 patients) found that MST produces a similar antidepressant effect to ECT (38). Furthermore, the optimal parameters of MST need to be determined.

As for other neurotherapeutic strategies, such as tDCS, DBS, or ECT, the main objective in investigating MST is to monitor the effects on neurocognition. The findings of this systematic review are that MST has little to no adverse neurocognitive effects, supporting the findings of an early study (47). However, the findings of the two included studies on the neurocognitive effects of MST were inconsistent (20, 21). Thus, more studies need to be performed to determine the neurocognitive effects of MST in schizophrenia. Interestingly, several clinical trials have shown non-convulsive electrotherapy to be effective for individuals suffering from schizophrenia (15) and TRD (48, 49) without associated adverse neurocognitive effects. However, no head-to-head studies have been published that compare the efficacy and safety of MST and non-convulsive electrotherapy in treating schizophrenia.

This systematic review is limited for the following reasons. First, only two open-label self-controlled studies (20, 21) with relatively small sample sizes were included. Second, a quantitative analysis could not be conducted because of the heterogeneity between the studies. Third, this systematic review has not been registered before the beginning of this systematic review. Finally, a high rate of discontinuation of MST for any reason was reported in both studies (20, 21), indicating the difficulty of treating individuals experiencing schizophrenia. In future clinical studies on adjunctive MST for schizophrenia, strategies need to be developed to address the problem of discontinuation.

## Conclusions

There is inconsistent evidence for MST-related adverse neurocognitive effects and preliminary evidence for the alleviation of psychotic symptoms in schizophrenia. RCTs with an optimal sample size need to be performed on the use of adjunctive MST for schizophrenia to confirm and extend these findings.

## Data Availability Statement

The original contributions presented in the study are included in the article/Supplementary Material, further inquiries can be directed to the corresponding author/s.

## Author Contributions

X-YZ and X-HY selected studies and extracted the data. WZ reviewed all the data and helped mediate disagreements. X-YZ, WZ, and D-BC wrote the first draft. All authors contributed to the interpretation of data and approved the final manuscript.

## Funding

This study was funded by the National Natural Science Foundation of China (82101609), Scientific Research Project of Guangzhou Bureau of Education (202032762), Science and Technology Program Project of Guangzhou (202102020658), the Science and Technology Planning Project of Liwan District of Guangzhou (202004034), Guangzhou Health Science and Technology Project (20211A011045), Guangzhou science and Technology Project of traditional Chinese Medicine and integrated traditional Chinese and Western medicine (20212A011018), China International Medical Exchange Foundation (Z-2018-35-2002), Guangzhou Clinical Characteristic Technology Project (2019TS67), science and Technology Program Project of Guangzhou (202102020658), and Guangdong Hospital Association (2019ZD06). The funders had no role in study design, data collection and analysis, decision to publish, or preparation of the manuscript.

## Conflict of Interest

The authors declare that the research was conducted in the absence of any commercial or financial relationships that could be construed as a potential conflict of interest.

## Publisher's Note

All claims expressed in this article are solely those of the authors and do not necessarily represent those of their affiliated organizations, or those of the publisher, the editors and the reviewers. Any product that may be evaluated in this article, or claim that may be made by its manufacturer, is not guaranteed or endorsed by the publisher.
