# Supplementary material for: Adjunctive Magnetic Seizure Therapy for Schizophrenia: A Systematic Review
Source: Front Psychiatry. 2022 Jan 10;12:813590. doi: 10.3389/fpsyt.2021.813590 (PMC8785398; doi:10.3389/fpsyt.2021.813590)
Supplement: Supplementary file 1 [file Table_1.docx]

**Supplemental Table 1.** GRADE analyses: adjunctive MST for schizophrenia

| **Design** | N (studies) | Risk of bias | Inconsistency | Indirectness | Imprecision | Publication bias | Large effect | Overall quality of evidence^a^ |
| --- | --- | --- | --- | --- | --- | --- | --- | --- |
| **Open-label self-controlled studies** | | | | | | | | |
| The improvement of psychotic symptoms | 16 (2) | Serious^b^ | No | No | Serious^c^ | Undetected | No | +/+/-/-; Low |
| Neurocognitive adverse events | 16 (2) | Serious^b^ | No | No | Serious^c^ | Undetected | No | +/+/-/-; Low |
| Discontinuation due to any reason | 16 (2) | Serious^b^ | No | No | Serious^d^ | Undetected | No | +/+/-/-; Low |
| Abbreviations: GRADE=Grading of Recommendations Assessment, Development, and Evaluation; MST=Magnetic Seizure Therapy.  ^a^GRADE Working Group grades of evidence: High quality=further research is very unlikely to change our confidence in the estimate of effect. Moderate quality=further research is likely to have an important impact on our confidence in the estimate of effect and may change the estimate. Low quality=further research is very likely to have an important impact on our confidence in the estimate of effect and is likely to change the estimate. Very low quality =we are very uncertain about the estimate. ^b^The two studies were single-group, before-after studies.  ^c^For continuous outcomes, N<400; For dichotomous outcomes, N<300. | | | | | | | | |
